# Early response to eptinezumab indicates high likelihood of continued response in patients with chronic migraine

**DOI:** 10.1186/s10194-022-01387-y

**Published:** 2022-02-21

**Authors:** Dawn C. Buse, Paul K. Winner, Larry Charleston, Joe Hirman, Roger Cady, Thomas Brevig

**Affiliations:** 1 Albert Einstein College of Medicine, Bronx, NY USA; 2 Vector Psychometric Group, LLC, Chapel Hill, NC USA; 3 Palm Beach Headache Center, West Palm Beach, FL USA; 4 Department of Neurology and Ophthalmology, Michigan State University College of Human Medicine, East Lansing, MI USA; 5 Pacific Northwest Statistical Consulting, Inc., Woodinville, WA USA; 6 Lundbeck LLC, IL Deerfield, USA; 7 H. Lundbeck A/S, Copenhagen, Denmark

**Keywords:** Eptinezumab, Chronic migraine, Migraine prevention, Clinical response

## Abstract

**Background:**

A clinical ability to describe the response trajectory of patients receiving preventive migraine treatment could expedite and improve therapeutic management decisions. This post hoc analysis of the PROMISE-2 study evaluated the consistency and predictive power of Month 1 treatment response on later response in patients with chronic migraine.

**Methods:**

PROMISE-2 was a double-blind, placebo-controlled trial that randomized adults with chronic migraine to eptinezumab 100 mg, 300 mg, or placebo administered IV every 12 weeks for up to 24 weeks (2 infusions over 6 study months). Migraine responder rates (MRRs) were calculated from monthly migraine days over 4-week intervals compared with baseline. Patients were grouped by MRR during Month 1 (< 25%, 25–< 50%, 50–< 75%, and ≥ 75%), with the number of subsequent study months (Months 2–6) with ≥50% and ≥ 75% MRR calculated in each subgroup. A similar analysis was conducted using Patient Global Impression of Change (PGIC) rating to define Month 1 subgroups (very much improved, much improved, minimally improved, and no change/worse) and rates of very much improved or much improved PGIC during Months 2–6.

**Results:**

In the eptinezumab 100 mg, 300 mg, and placebo groups, respectively, 194/356 (54.5%), 212/350 (60.6%), and 132/366 (36.1%) patients were ≥ 50% migraine responders during Month 1. More eptinezumab-treated patients were ≥ 75% migraine responders (100 mg, 110/356 [30.9%]; 300 mg, 129/350 [36.9%]; placebo, 57/366 [15.6%]) and more placebo-treated patients were < 25% migraine responders (eptinezumab 100 mg, 103/356 [28.9%]; 300 mg, 80/350 [22.9%]; placebo, 153/366 [41.8%]). Among patients who achieved ≥75% migraine response in Month 1, more than one-third attained ≥75% migraine response for all 5 subsequent study months and more than two-thirds achieved ≥75% migraine response for ≥3 months. More than two-thirds of those in the very much improved (PGIC) subgroup at Month 1 were much or very much improved for all 5 subsequent months.

**Conclusions:**

In this post hoc analysis of data from PROMISE-2, more eptinezumab-treated than placebo-treated patients were early (Month 1) responders, and most early responders went on to achieve a high level of response for at least half of the 24-week treatment period. Potential for later response in early non-responders was also observed.

**Trial registration:**

ClinicalTrials.gov identifier: NCT02974153; registered November 23, 2016.

## Introduction

For patients living with chronic migraine [1], preventive treatment that effectively reduces the number and burden of migraine attacks has the potential to change lives and daily functioning. By reducing the number of migraine days and associated disability, potentially lessening the likelihood of developing comorbidities, and decreasing the direct and indirect costs associated with chronic migraine [1, 2], preventive agents can considerably reduce the burden of this disorder.

The American Headache Society recommends that clinicians help patients establish realistic expectations regarding the anticipated benefits of prescribed preventive migraine medications [3]. However, the recent introduction of calcitonin gene-related peptide (CGRP) antagonists has changed the treatment paradigm for patients requiring migraine prevention. Treatments targeting CGRP typically have a more rapid onset of action [4–18] with no or minimal interim period between treatment initiation and full onset of efficacy, and these attributes have the potential to decisively change patient management. Thus, the ability of clinicians to describe the response trajectory of a particular preventive treatment agent at an early stage could accelerate management decisions related to continuation of that specific treatment, and interim use of acute medication.

Eptinezumab (Vyepti™, Lundbeck Seattle BioPharmaceuticals, Inc., Bothell, WA, USA), is an intravenously administered monoclonal antibody targeting the CGRP ligand and is approved for the preventive treatment of migraine in adults [19]. In the pivotal PROMISE-2 study of patients with chronic migraine, eptinezumab—at the approved doses of 100 mg and 300 mg—significantly reduced migraine frequency, was well tolerated, and had an acceptable safety profile [16]. The onset of response was rapid, with a > 50% reduction in migraine prevalence on the day after dosing in the eptinezumab treatment groups. During the first month (Weeks 1–4) of the study, approximately a third of eptinezumab-treated patients had a ≥ 75% migraine response [16]. In addition, 45% and 59% of patients receiving eptinezumab 100 mg and 300 mg, respectively, reported “much” or “very much” improvement through Weeks 1–4, based on the Patient Global Impression of Change (PGIC) [20].

The objective of this post hoc analysis of the PROMISE-2 study was to evaluate the general consistency and predictive power of treatment response—defined by reduction in the frequency of monthly migraine days (MMDs) and by patient perception of change in disease during the first month of eptinezumab treatment—on later response outcomes in patients with chronic migraine.

## Methods

### Study design, patients, and interventions

PROMISE-2 (NCT02974153) was a randomized, double-blind, placebo-controlled, parallel-group, phase 3 clinical trial that evaluated the preventive efficacy, tolerability, and safety of eptinezumab in adults with chronic migraine [16]. The study was conducted in accordance with the International Conference on Harmonisation Good Clinical Practice guidelines, the principles of the Declaration of Helsinki, and all applicable local regulatory requirements. The study was approved by the independent ethics committee or institutional review board for each study site.

Detailed methodology has been published [16]. In brief, eligibility criteria were an age of 18–65 years, a diagnosis of migraine at or before age 50 years, a history of chronic migraine for at least 12 months before screening, completion of a headache electronic diary (eDiary) on at least 24 of the 28 days during the screening period, and experiencing 15–26 headache days and at least 8 migraine days during the screening period. Key exclusion criteria were presence of a confounding pain disorder or clinically significant pain syndromes, and history or diagnosis of a headache or migraine disorder that did not meet Section 1.3 criteria for chronic migraine per the 2013 International Classification of Headache Disorders, 3rd edition, beta version (ICHD-3β) [21]. This diagnoses was also made by ICHD beta criteria. This may not be an essential correction as ICHD-3 criteria were not published until 1, 2018. All patients enrolled in the study provided written informed consent before their participation.

Eligible patients were randomized to intravenous eptinezumab 100 mg, eptinezumab 300 mg, or placebo at 28–30 days after the screening visit and were administered the first dose of study drug on Day 0, within 8 days after the randomization visit. A second dose was administered on Day 84 (Week 12), with 24 weeks included in the study treatment period. Patients completed a daily eDiary from the time of screening through Week 24 to capture daily headache episodes and migraine attacks (defined per ICHD-3β).

### Outcome measures

The mean frequencies of MMDs and migraine responder rates (MRRs) were calculated from eDiary data over 4-week intervals (Weeks 1–4; 5–8; 9–12; 13–16; 17–20; 21–24). A migraine responder was classified as a patient who achieved the specified reduction (< 25%, 25–< 50%, 50–< 75%, and ≥ 75%) in MMDs. The baseline frequency of migraine days was then compared to the migraine frequency in the 4-week intervals.

Patients also completed patient-reported outcome measures during the study, including the PGIC, which were evaluated at monthly intervals. The PGIC asked a single question concerning the patient’s impression of the overall change in their disease status since the start of the study, which was rated on a 7-category scale ranging from “very much improved” to “very much worse.”

For this post hoc analysis, patients were grouped by Month 1 response category (i.e., < 25%, 25–< 50%, 50–< 75%, and ≥ 75% MRR), and the number of subsequent study months with ≥50% and ≥ 75% MRR was calculated for each subgroup. The predictive ability of Month 1 PGIC ratings (very much improved, much improved, minimally improved, and no change/worse) were evaluated to determine which patients returned a PGIC response of much or very much improved at Month 6. Patients were also grouped by Month 1 PGIC response to assess the frequency of PGIC responses of much or very much improved during the subsequent study months.

### Statistical analyses

The full analysis set comprised all patients who received study medication. For PGIC calculations, only patients with responses at all specified timepoints were included. All analyses were conducted with SAS software (SAS Institute, Inc., Cary, NC, USA) version 9.2 or higher.

Monthly results (MMDs and MRRs) were calculated using the 4-week eDiary data intervals. If the eDiary was completed for at least 21 but less than 28 days, the observed frequency was normalized to the full 28-day period. For patients with eDiary data for less than 21 of 28 days, the results were proportionally weighted based on the observed data plus data from the previous 4-week interval.

By comparing the baseline frequency of migraine days to the migraine frequency in each 4-weekly interval, it was possible to calculate changes; by determining a percent change from baseline, the migraine responder status (< 25%, 25–< 50%, 50–< 75%, and ≥ 75%) could be obtained. Initial responses (MRR and PGIC) were based on Month 1 data; response during the subsequent study months was based upon data reported during Months 2–6 (i.e., 5–8, 9–12, 13–16, 17–20, and 21–24 weeks).

## Results

A total of 1072 adults with chronic migraine participated in PROMISE-2 (mean age, 40.5 years; 88.2% female; 91.0% white) [16]. This included 431 (40.2%) with medication-overuse headache. Of the 1072 patients treated, 356 (33.2%) received eptinezumab 100 mg, 350 (32.6%) received eptinezumab 300 mg, and 366 (34.1%) received placebo.

### Monthly migraine responder rates

**Fig. 1:**
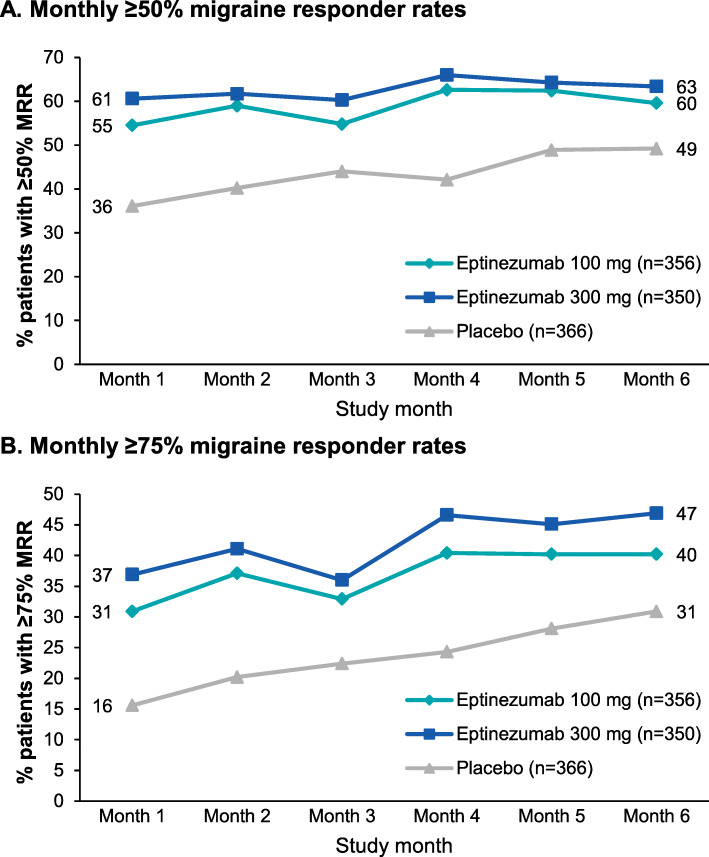
Monthly migraine responder^a^ rates: (**A**) ≥50% and (**B**) ≥75%. ^a^Migraine response was defined as a reduction in monthly migraine days. MRR, migraine responder rate

Over 6 study months, more patients treated with eptinezumab 100 mg or 300 mg were ≥ 50% or ≥ 75% migraine responders compared with patients receiving placebo (Fig. 1). In eptinezumab-treated patients, ≥50% migraine responder rates were generally consistent from Month 1 (100 mg, 194/356, 54.5%; 300 mg, 212/350, 60.6%) to Month 6 (100 mg, 212/356, 59.6%; 300 mg, 222/350, 63.4%), with placebo patients showing a slight increase over time (Month 1, 132/366, 36.1%; Month 6, 180/366, 49.2%). The ≥75% migraine responder rates increased for all treatment arms from Month 1 (100 mg, 110/356, 30.9%; 300 mg, 129/350, 36.9%; placebo, 57/366, 15.6%) to Month 6 (100 mg, 143/356, 40.2%; 300 mg, 164/350, 46.9%; placebo, 113/366, 30.9%).

### Migraine response during month 1

In the eptinezumab 100 mg, 300 mg, and placebo groups, respectively, 194/356 (54.5%), 212/350 (60.6%), and 132/366 (36.1%) patients were ≥ 50% migraine responders (i.e., had a ≥ 50% reduction in MMDs) during Month 1 (Table 1). Although rates of 50–< 75% migraine response were similar across treatment groups (eptinezumab 100 mg, 84/356 [23.6%]; 300 mg, 83/350 [23.7%]; placebo, 75/366 [20.5%]), more eptinezumab-treated than placebo-treated patients were ≥ 75% migraine responders (100 mg, 110/356 [30.9%]; 300 mg, 129/350 [36.9%]; placebo, 57/366 [15.6%]). Indeed, in the eptinezumab groups, most patients who were ≥ 50% migraine responders were also ≥75% migraine responders (100 mg, 110/194 [56.7%]; 300 mg, 129/212 [60.8%]; placebo, 57/132 [43.2%]). Conversely, more placebo-treated than eptinezumab-treated patients were 25–< 50% migraine responders (100 mg, 59/356 [16.6%]; 300 mg, 58/350 [16.6%]; placebo, 81/366 [22.1%]) or < 25% migraine responders (103/356 [28.9%]; 80/350 [22.9%]; 153/366 [41.8%], respectively).

**Table 1:** MRR and PGIC at Month 1

	Eptinezumab 100 mg	Eptinezumab 300 mg	Placebo
MRR			
*n*	356	350	366
≥ 75%	110 (30.9)	129 (36.9)	57 (15.6)
50–< 75%	84 (23.6)	83 (23.7)	75 (20.5)
25–< 50%	59 (16.6)	58 (16.6)	81 (22.1)
< 25%	103 (28.9)	80 (22.9)	153 (41.8)
PGIC			
n*	328	329	330
Very Much Improved	41 (12.5)	81 (24.6)	27 (8.2)
Much Improved	110 (33.5)	112 (34.0)	80 (24.2)
Minimally Improved	103 (31.4)	76 (23.1)	82 (24.8)
No Improvement^†^	74 (22.6)	60 (18.2)	141 (42.7)

*Only patients with data at Month 1 and Month 6 are included

^†^No improvement includes “no change,” “minimally worse,” “much worse,” and “very much worse”

MRR, migraine response rate; PGIC, Patient Global Impression of Change

During Month 1, ≥75% migraine responders demonstrated a mean (standard deviation) reduction in MMDs of approximately 90% (eptinezumab 100 mg, − 89.0% [8.34]; 300 mg, − 90.8% [8.55]; placebo, − 87.3% [8.58]). MMD reductions in the 50–< 75%, 25–< 50%, and < 25% migraine responder subgroups were approximately 62%, 36%, and 4%, respectively.

Baseline characteristics according to Month 1 migraine responses are shown in Table 2. No notable differences in demographic or clinical variables were observed between patients in each quartile, suggesting that none of these baseline factors influenced migraine response during Month 1.

**Table 2:** Baseline demographics and characteristics by Month 1 migraine response and treatment

	≥75%			50–< 75%			25–< 50%			< 25%		
	100 mg (*n* = 110)	300 mg (*n* = 129)	Placebo (*n* = 57)	100 mg (*n* = 84)	300 mg (*n* = 83)	Placebo (*n* = 75)	100 mg (*n* = 59)	300 mg (*n* = 58)	Placebo (*n* = 81)	100 mg (*n* = 103)	300 mg (*n* = 80)	Placebo (*n* = 153)
Mean age, years (SD)	42.1 (11.4)	41.6 (10.1)	40.5 (12.3)	41.4 (11.2)	40.8 (10.7)	39.3 (10.9)	37.9 (11.3)	40.2 (10.0)	39.6 (10.2)	41.4 (12.5)	40.9 (10.8)	39.5 (11.7)
Sex: female, *n* (%)	92 (83.6)	115 (89.1)	49 (86.0)	76 (90.5)	75 (90.4)	66 (88.0)	50 (84.7)	56 (96.6)	72 (88.9)	89 (86.4)	68 (85.0)	138 (90.2)
Race, *n* (%)												
White	104 (94.5)	121 (93.8)	51 (89.5)	81 (96.4)	78 (94.0)	59 (78.7)	56 (94.9)	51 (87.9)	74 (91.4)	91 (88.3)	72 (90.0)	137 (89.5)
Black or African American	6 (5.5)	6 (4.7)	4 (7.0)	3 (3.6)	3 (3.6)	15 (20.0)	3 (5.1)	7 (12.1)	6 (7.4)	9 (8.7)	7 (8.8)	13 (8.5)
Other	0	2 (1.6)	2 (3.5)	0	2 (2.4)	1 (1.3)	0	0	1 (1.2)	3 (2.9)	1 (1.3)	3 (2.0)
Mean BMI, kg/m^2^ (SD)	26.1 (4.1)	26.3 (4.6)	27.2 (5.0)	25.6 (5.2)	26.3 (5.0)	27.1 (5.4)	27.2 (5.5)	25.0 (4.3)	27.0 (6.0)	27.0 (5.3)	27.1 (6.0)	26.9 (5.7)
Mean age at diagnosis, years (SD)	23.2 (10.4)	22.5 (9.3)	22.2 (10.5)	22.1 (10.2)	22.0 (9.5)	23.3 (9.1)	24.1 (12.2)	21.4 (9.1)	22.8 (9.7)	22.0 (10.3)	21.8 (9.4)	22.3 (10.4)
Mean duration of migraine diagnosis, years (SD)	18.9 (12.4)	19.1 (11.5)	18.3 (13.5)	19.3 (11.4)	18.8 (11.2)	16.0 (10.4)	13.8 (10.1)	18.7 (12.1)	16.8 (10.0)	19.4 (13.3)	19.1 (11.6)	17.2 (12.3)
Mean duration of chronic migraine, years (SD)	10.4 (11.9)	11.2 (11.2)	12.3 (12.4)	10.3 (10.8)	13.5 (12.5)	9.5 (9.8)	10.7 (11.0)	12.9 (10.6)	10.4 (9.2)	14.3 (12.4)	12.6 (10.0)	13.1 (11.5)
Mean baseline MHDs (SD)	19.6 (2.6)	20.0 (3.3)	21.1 (3.0)	20.4 (3.1)	20.4 (3.0)	20.7 (2.8)	20.4 (3.0)	20.8 (3.3)	20.5 (3.0)	21.1 (3.5)	21.0 (3.3)	20.4 (3.1)
Mean baseline MMDs (SD)	15.4 (4.0)	14.8 (4.6)	16.2 (4.7)	16.2 (4.7)	16.2 (4.9)	16.7 (4.0)	16.5 (4.5)	17.2 (4.3)	16.6 (4.4)	16.5 (5.2)	17.2 (4.8)	15.8 (4.8)
MOH diagnosis, n (%)	45 (40.9)	54 (41.9)	23 (40.4)	33 (39.3)	32 (38.6)	28 (37.3)	22 (37.3)	28 (48.3)	33 (40.7)	39 (37.9)	33 (41.3)	61 (39.9)
Mean HIT-6 total score (SD)	64.1 (4.9)	64.4 (5.1)	63.8 (6.3)	65.6 (4.9)	65.1 (4.7)	64.8 (5.4)	66.1 (4.2)	66.5 (5.3)	65.3 (5.0)	64.8 (5.4)	65.1 (4.7)	64.9 (5.4)

BMI, body mass index; HIT-6, 6-item Headache Impact Test; MHDs, monthly headache days; MMDs, monthly migraine days; MOH, medication-overuse headache; SD, standard deviation

### MRR during subsequent study months

**Fig. 2:**
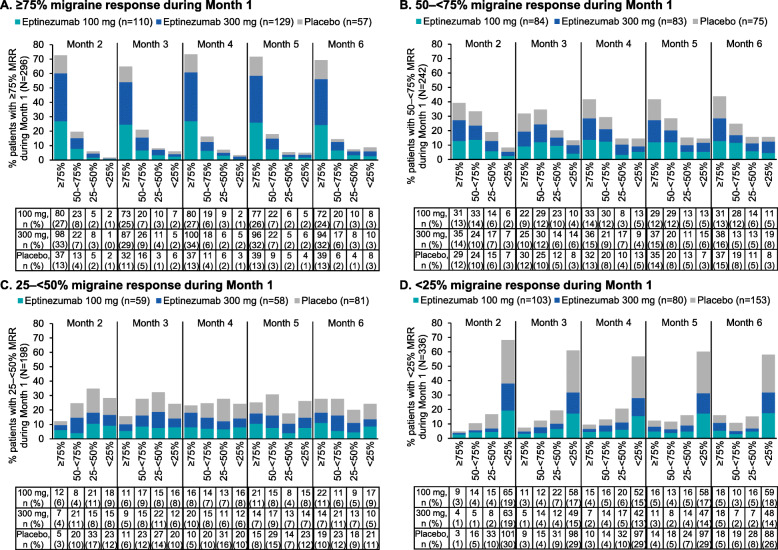
Monthly response according to Month 1 migraine response^a^ category: (**A**) ≥75%, (**B**) 50–< 75%, (**C**) 25–< 50%, (**D**) < 25%. ^a^Migraine response was defined as a percentage reduction in monthly migraine days. MRR, migraine responder rate

**Fig. 3:**
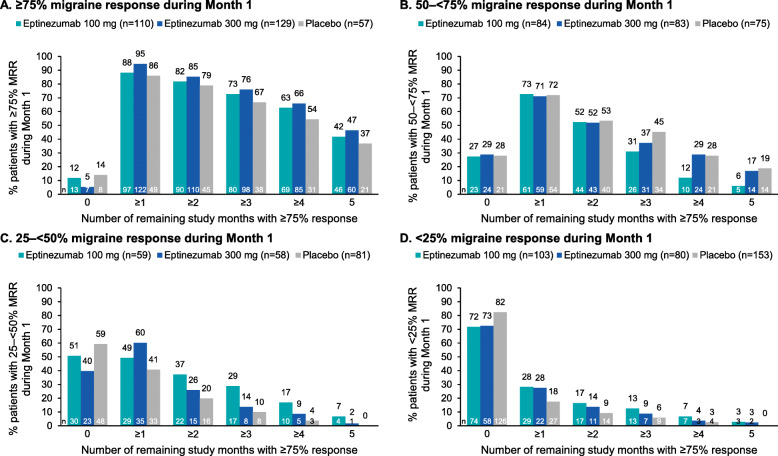
Frequency of monthly ≥75% migraine response according to Month 1 migraine response^a^ category: (**A**) ≥75%, (**B**) 50–< 75%, (**C**) 25–< 50%, (**D**) < 25%**.** ^a^Migraine response was defined as a reduction in monthly migraine days. MRR, migraine responder rate

**Fig. 4:**
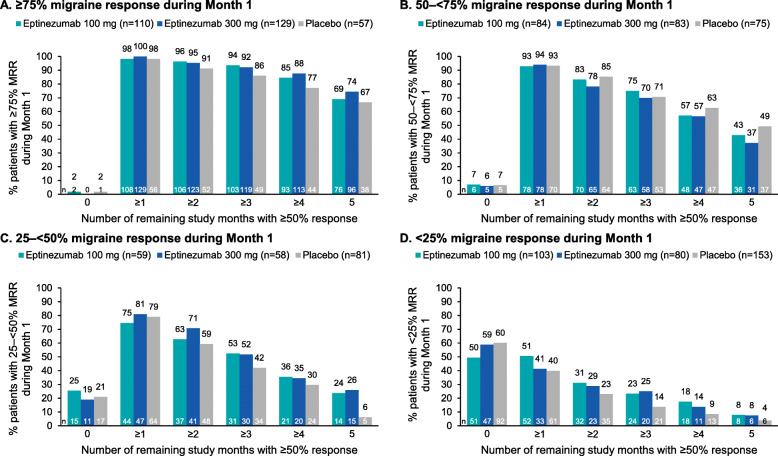
Frequency of monthly ≥50% migraine response according to Month 1 migraine response^a^: (**A**) ≥75%, (**B**) 50–< 75%, (**C**) 25–< 50%, (**D**) < 25%. ^a^Migraine response was defined as a percentage reduction in monthly migraine days. MRR, migraine responder rate

Response trajectories based on Month 1 response are shown in Fig. 2. Consistency of Month 1 data during the subsequent study months, according to response group, is shown in Figs. 3 and 4.

Most patients who achieved ≥75% migraine response during Month 1 subsequently experienced ≥75% migraine response over each study month (Month 2, 215/296, 72.6%; Month 3, 192/296, 64.9%; Month 4, 217/296, 73.3%; Month 5, 212/296, 71.6%; Month 6, 205/296, 69.3%) (Fig. 2 A). Among patients who achieved ≥75% migraine response in Month 1, more than one-third (127/296 [42.9%]) continued to attain ≥75% migraine response for all 5 subsequent study months (eptinezumab 100 mg, 46/110 [41.8%]; 300 mg, 60/129 [46.5%]; placebo, 21/57 [36.8]%) (Fig. 3 A). More than two-thirds of Month 1 ≥ 75% responders achieved ≥75% migraine response for ≥3 subsequent months (80/110 [72.7%]; 98/129 [76.0%]; and 38/57 [66.7%], respectively) (Fig. 3 A). In addition, among the patients who achieved ≥75% migraine response in Month 1, nearly all (293/296 [99.0%]) had at least 1 month of ≥50% migraine response during the subsequent 5 months, and more than two-thirds (210/296 [70.9%]) had a ≥ 50% migraine response for all 5 subsequent study months (100 mg, 76/110 [69.1%]; 300 mg, 96/129 [74.4%]; placebo, 38/57 [66.7%]) (Fig. 4 A).

Most patients who achieved 50–< 75% migraine response during Month 1 subsequently experienced ≥50% migraine response over each study month (Month 2, 176/242, 72.7%; Month 3, 161/242, 66.5%; Month 4, 172/242, 71.1%; Month 5, 170/242, 70.2%; Month 6, 166/242, 68.6%) (Fig. 2 B). During Months 2 and 3, the proportion of ≥75% and 50–75% response was similar; during Months 4 through 6, more patients were ≥ 75% responders than ≥50% responders. Among patients who achieved 50–< 75% migraine response in Month 1, more than one-third (104/242 [43.0%]) achieved ≥50% migraine response for all 5 subsequent study months (100 mg, 36/84 [42.9%]; 300 mg, 31/83 [37.3%]; placebo, 37/75 [49.3%]) and more than two-thirds achieved ≥50% migraine response for ≥3 months (63/84 [75.0%]; 58/83 [69.9%]; and 53/75 [70.7%], respectively) (Fig. 4 B).

Three times as many eptinezumab-treated patients who were 25–< 50% migraine responders in Month 1 achieved ≥50% migraine response for all 5 subsequent study months compared with placebo (eptinezumab 100 mg, 14/59 [23.7%]; 300 mg, 15/58 [25.9%]; placebo, 5/81 [6.2%]) (Fig. 4 C). Overall, about half of patients in this Month 1 response group (95/198 [48.0%]) still achieved ≥3 study months with ≥50% migraine response (100 mg, 31/59 [52.5%]; 300 mg, 30/58 [51.7%]; placebo, 34/81 [42.0%]).

Among patients with < 25% migraine response during Month 1, 8/103 (7.8%), 6/80 (7.5%), and 6/153 (3.9%) of patients in the eptinezumab 100 mg, 300 mg, and placebo groups, respectively, achieved ≥50% migraine response for all 5 subsequent study months, while 52/103 (50.5%), 33/80 (41.3%), and 61/153 (39.9%), respectively, achieved ≥1 subsequent study month with ≥50% migraine response (Fig. 4 D). More eptinezumab-treated than placebo-treated patients with < 25% migraine response during Month 1 achieved ≥3 subsequent months of ≥50% migraine response (eptinezumab 100 mg, 24/103 [23.3%]; 300 mg, 20/80 [25.0%]; placebo, 21/153 [13.7%]).

### PGIC ratings at month 1 and PGIC response during the subsequent study months

Of patients with PGIC data available at both Month 1 and Month 6, 151/328 (46.0%) of those who received eptinezumab 100 mg and 193/329 (58.7%) who received eptinezumab 300 mg indicated that their condition was very much or much improved at Month 1 (Table 1). In contrast, 107/330 (32.4%) of those who received placebo were very much or much improved at Month 1. Of the remaining placebo-treated patients, 82/330 (24.8%) were minimally improved, and 141/330 (42.7%) indicated no improvement at Month 1.

PGIC ratings of *very much improved* or *much improved* at Month 1 were predictive of PGIC ratings at Month 6. More than 80% of the subgroup of patients who were *very much improved* at Month 1 were *very much improved* or *much improved* at Month 6 (82.9%, 86.4%, and 81.5% for eptinezumab 100 mg, eptinezumab 300 mg, and placebo, respectively). For the subgroup of patients who were *much improved* at Month 1, rates of *very much improved* or *much improved* at Month 6 were 71.8%, 75.9%, and 66.3%, respectively.

**Fig. 5:**
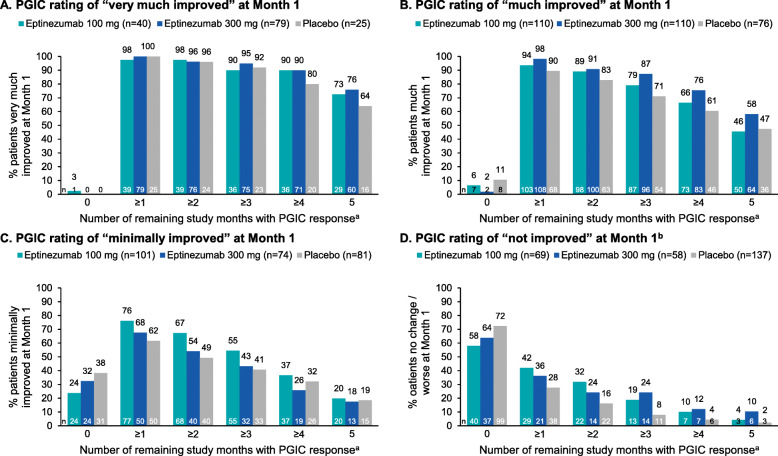
Frequency of PGIC response^a^ according to PGIC rating at Month 1: (**A**) very much improved, (**B**) much improved, (**C**) minimally improved, (**D**) no improvement. ^a^PGIC response defined as rating of “much improved” or “very much improved.” ^b^Includes patients reporting “no change,” “minimally worse,” “much worse,” and “very much worse.” Analysis conducted in patients with PGIC data at all time points. PGIC, Patient Global Impression of Change

Of patients with PGIC data at all timepoints, more than 90% of the *very much improved* and *much improved* subgroups during Month 1 had at least 1 subsequent month of the same response level. More than two-thirds of those in the *very much improved* subgroup were *very much improved* or *much improved* for all 5 subsequent months, and approximately half of those in the *much improved* subgroup were *very much improved* or *much improved* for all 5 subsequent study months (Fig. 5).

## Discussion

The ability to assess early-stage responses to a preventive migraine treatment and predict the subsequent response trajectory would better equip clinicians to manage patient expectations and improve therapeutic decision-making. In this post hoc analysis of the PROMISE-2 study in patients with chronic migraine, we evaluated the consistency and predictive ability of migraine response during Month 1 on later outcomes. Our data confirmed that more eptinezumab-treated than placebo-treated patients were ≥ 50% and ≥ 75% migraine responders during the entire study period, including Month 1, and revealed that an early response was generally predictive of a sustained response, with many patients experiencing consistent benefits during the subsequent months.

The aim of the analysis was to highlight the consistency and general predictive ability of eptinezumab preventive efficacy, which biologically and clinically has shown rapid onset of action [22, 23]; this, in turn, is expected to allow earlier clinical decision-making regarding future patient care. Eptinezumab treatment resulted in greater rates of migraine and PGIC response than placebo, and our data indicate that Month 1 response to treatment was predictive of response throughout the study, with most eptinezumab-treated patients who achieved Month 1 migraine response continuing to respond at the same level or higher for at least half of the 24-week treatment period. Although some other new migraine preventives have demonstrated an early onset of effect, this is, to our knowledge, the first analysis to describe the response trajectory based on the first month of treatment.

The American Headache Society recommends that clinicians help patients establish realistic expectations regarding the anticipated benefits of prescribed preventives, including effects on migraine and headache frequency, attack duration, attack severity, migraine-related disability, psychological distress, acute treatment response, functioning in important areas of life, and/or health-related quality of life [3]. Identification of early non-response is also an opportunity for clinicians to advance patient management in a timely manner. The results of the current analysis suggest that patients initiating eptinezumab may be able to recognize their response trajectories (with respect to migraine frequency and PGIC) earlier than patients beginning oral preventives (1 month vs 2–12 months, respectively) [3]. This early onset and consistency of response is not unexpected, given the pharmacokinetics and mechanism of action of eptinezumab. Intravenous administration of eptinezumab ensures 100% bioavailability, with maximum plasma concentration by the end of the infusion (i.e., ~ 30 min) [24]. In addition, eptinezumab has a highly potent and selective binding profile for the CGRP ligand [22] and a terminal elimination half-life of ~ 27 days [19]. These attributes are responsible for the early onset of efficacy (preventive efficacy on Day 1 after dosing [25] and symptomatic efficacy after 2 h when administered during a migraine attack [26]) previously reported for eptinezumab, the sustained effectiveness observed over 12 weeks post-dose [16, 27, 28], and the persistent or increased effects reported with additional dosing [29].

The high rates of response to eptinezumab during Month 1 and the maintenance and consistency of the subsequent response profile could potentially help to provide guidance and/or a framework for management decisions for patients with chronic migraine. For clinicians, these decisions include whether to continue with preventive treatment, the necessity for interim prescription of acute medication, and recommendations for lifestyle modifications and bio-behavioral training. For patients, prediction of response may simplify future planning for their professional and social lives and provide more realistic treatment expectations according to the individuality of migraine phenotypes and the burden of disease. These results will likely also inspire hope and optimism in patients, which may have the effect of improving psychological symptoms and reducing migraine-related distress, although formal effects on health-related quality of life remain to be confirmed. To fully achieve treatment response prediction, additional studies are needed to explore relationships between the early onset of effect and other outcome measures, such as the Migraine Disability Assessment, 6-item Headache Impact Test, 36-item Short Form Health Survey, Migraine Interictal Burden Scale, Migraine-Specific Quality of Life Questionnaire, Patient-Identified Most Bothersome Symptom, acute headache medication use, and psychologic symptomatology. These additional studies should also account for the inherent month-to-month variability of migraine frequency as well as the administration of multiple doses, which were not explored in the current analysis.

It is clear that early response with eptinezumab has predictive value for subsequent response. However, our data also indicated that some patients who were early non-responders at Month 1 could still become responders in subsequent time periods. Approximately 50% and 25% of eptinezumab-treated patients with 25–< 50% and < 25% migraine response during Month 1, respectively, achieved ≥50% migraine response for at least half of the 6-month treatment period (vs 42% and 14% with placebo, respectively). This indicates the importance of not halting or switching eptinezumab treatment too early and underscores the need for appropriate interim migraine management, as well as management of patient education and expectation.

Limitations of this study include the post hoc design of the analyses, which necessitates additional studies to confirm these findings and to identify the predictive value of early response with respect to other outcomes. Notably, due to the heterogeneous nature of migraine [30], the MMD measure has inherent month-to-month variability, and we consider that this may have reduced the Month 1 predictive ability. That is, for patients who were naturally experiencing relative improvements during the course of their chronic migraine, smaller therapeutic benefit (impact of eptinezumab on MMDs) would be observed, whereas for patients experiencing relative worsening of migraine at the time of the study, the beneficial impact of eptinezumab may have been overemphasized. As in many previous clinical trials in migraine patients, there was a high placebo response rate in this phase 3 study; however, effectiveness in the placebo group remained consistently lower than that of eptinezumab in terms of response rates and PGIC improvement. Finally, based on the demographic characteristics that were reported in the full study population (i.e., predominantly white and non-Hispanic) [16], the results may not be generalizable to all patients with chronic migraine, with further research warranted in more racially and ethnically diverse patient groups.

## Conclusion

In this post hoc analysis of data from PROMISE-2, more eptinezumab-treated than placebo-treated patients were ≥ 75% MRR responders during Month 1. The majority of eptinezumab-treated patients who achieved a migraine response during Month 1 went on to achieve the same or a higher level of response for at least half of the entire 24-week treatment period. However, the potential for later response in early eptinezumab non-responders was also observed. These findings could help clinicians and patients set realistic treatment expectations and accelerate appropriate management decisions.

## Data Availability

In accordance with EFPIA’s and PhRMA’s “Principles for Responsible Clinical Trial Data Sharing” guidelines, Lundbeck is committed to responsible sharing of clinical trial data in a manner that is consistent with safeguarding the privacy of patients, respecting the integrity of national regulatory systems, and protecting the intellectual property of the sponsor. The protection of intellectual property ensures continued research and innovation in the pharmaceutical industry. Deidentified data is available to those whose request has been reviewed and approved through an application submitted to https://www.lundbeck.com/global/our-science/clinical-data-sharing.

## Abbreviations

CGRP: Calcitonin gene-related peptide
eDiary: Electronic diary
ICHD-3β: International Classification of Headache Disorders, 3rd edition, beta version
MMDs: Monthly migraine days
MRR: Migraine responder rate
PGIC: Patient Global Impression of Change
